# Current status and factors influencing clinical nurses’ attitudes toward information security: a cross-sectional survey

**DOI:** 10.1590/1980-220X-REEUSP-2025-0391en

**Published:** 2026-03-27

**Authors:** Bai Xue, Xue Yurui, Guo Shuping, Ji Shuangdui, Zheng Donglian, Ma Fuzhen, Yin Changqi

**Affiliations:** 1 Ningxia Medical University, School of Nursing, Ningxia, China.; 2 General Hospital of Ningxia Medical University, Department of Nursing, Ningxia, China.; 3 General Hospital of Ningxia Medical University, Department of Cardiac Macrovascular Surgery, Ningxia, China.; 4 General Hospital of Ningxia Medical University, Department of Orthopaedic Trauma, Ningxia, China.

**Keywords:** Nurse Clinicians, Nursing Informatics, Computer Security, Attitude, Enfermeiros Clínicos, Informática em Enfermagem, Segurança Computacional, Atitude

## Abstract

**Objective:**

To explore the prevailing information security attitudes among clinical nurses and examine the factors influencing these attitudes.

**Method:**

In November and December 2024, a convenience sample method was employed to select 756 clinical nurses from ten public hospitals ranking second or above in five cities of the Ningxia Hui Autonomous Region as study participants. The survey utilized a nursing information competency evaluation scale, a general information questionnaire, and a questionnaire assessing nurses’ attitudes toward information security.

**Results:**

The total score for the information security attitude of 756 clinical nurses was (95.0; 87.0–104.0). Spearman’s correlation analysis indicated a significant positive correlation between nursing information competence and the clinical nurses’ information security attitude (r = 0.519; P < 0.05). The main results of the multiple regression were shown, with coefficients, confidence intervals, adjusted R^2^ = 0.329, and the list of retained predictors (age, hospital level, training offered, information literacy).

**Conclusion:**

This study revealed a moderately high level of information security attitudes among clinical nurses. Age, lack of prior training, and lower informatics competence were key factors associated with less favorable attitudes. Therefore, implementing targeted interventions is recommended to further strengthen security awareness and promote safe practices across nursing settings.

## RESUMO

**Objetivo:**

Explorar as atitudes predominantes em relação à segurança da informação entre enfermeiros clínicos e examinar os fatores que influenciam essas atitudes.

**Método:**

Em novembro e dezembro de 2024, um método de amostragem por conveniência foi empregado para selecionar 756 enfermeiros clínicos de dez hospitais públicos classificados em segundo lugar ou acima, em cinco cidades da Região Autônoma de Ningxia Hui, como participantes do estudo. A pesquisa utilizou uma escala de avaliação de competências em informação de enfermagem, um questionário de informações gerais e um questionário que avaliava as atitudes dos enfermeiros em relação à segurança da informação.

**Resultados:**

A pontuação total para a atitude em relação à segurança da informação de 756 enfermeiros clínicos foi de (95,0;87,0–104,0). A análise de correlação de Spearman indicou uma correlação positiva significativa entre a competência em informação de enfermagem e a atitude de segurança da informação dos enfermeiros clínicos (r = 0,519; P < 0,05). Os principais resultados da regressão múltipla, com coeficientes, intervalos de confiança e R^2^ ajustado = 0,329, e a lista de preditores retidos (idade, nível do hospital, treinamento oferecido, alfabetização informacional) foram apresentados.

**Conclusão:**

Este estudo revelou um nível moderadamente alto de atitudes em relação à segurança da informação entre enfermeiros clínicos. A idade, a falta de treinamento prévio e a menor competência em informática foram fatores-chave associados a atitudes menos favoráveis. Portanto, recomenda-se a implementação de intervenções direcionadas a fortalecer ainda mais a conscientização sobre segurança e promover práticas seguras em todos os ambientes de enfermagem.

## INTRODUCTION

With the advancement of information technology, Chinese hospitals are actively developing information systems and mobile health applications. While improving service efficiency, this has also underscored the security risks associated with patient data. Research indicates that approximately 70% of healthcare data breaches originate from inadequate security awareness among medical personnel^(1)^. Between 2009 and 2020, a total of 3,705 reported healthcare data breaches worldwide affected nearly 81.72% of the US population^(2)^, highlighting the criticality of this issue. Numerous studies suggest that the primary source of healthcare information security issues is inadequate security awareness and behavioral lapses among internal personnel in medical institutions^(3,4)^. Nursing information security, as a fundamental element of healthcare information systems, encompasses extensive amounts of confidential personal health data and essential medical information. Security incidents not only pose risks of intensifying doctor-patient conflicts, reducing the quality of nursing care, and adversely affecting patient experiences, but may also provoke negative public discourse and potentially endanger patient-health and safety^(5,6)^. Clinical nurses, as essential professionals directly responsible for handling, collecting, and managing patient information in clinical practice, serve a vital role within information security protection frameworks^(7,8)^. Their attitudes toward information security demonstrate not only individual dedication to security protocols but also a significant impact on the management and safeguarding of sensitive information^(9)^. International research has concentrated on nurses’ attitudes, behaviors, and competencies regarding information security, examining prevention strategies for the leakage, tampering, and annihilation of nursing information from technological and managerial control perspectives^(7)^. Clinical nurses’ attitudes toward information security reliably predict their information security behaviors^(9,10)^. However, domestic research is still in its early stages, focusing primarily on information management skills, with limited investigation into the current state of clinical nurses’ attitudes toward information security and the factors that influence them. To date, only initial investigations into the information security awareness of healthcare personnel have been conducted, such as the study by Guo and Xie^(11)^. Existing evidence suggests that clinical nurses frequently demonstrate inadequate risk awareness in information security practices, exhibit complacency toward safety protocols, and engage in behaviors such as indiscriminate account sharing and failure to log out of systems promptly^(12,13)^. They exhibit insufficient cognizance of safeguarding sensitive information, leading to non-compliant practices in the storage and transmission of patient data. Moreover, they fail to acknowledge the security risks posed by emerging technologies and rely excessively on default system configurations. As a key part of China’s western region, Ningxia shows certain deficiencies in promoting awareness of nursing information security and in implementing relevant systems^(14)^. Therefore, assessing the present attitudes toward information security among clinical nurses in this region and the factors influencing them is of substantial practical importance. This study seeks to thoroughly evaluate the current state of clinical nurses’ attitudes toward information security, analyze the primary influencing factors, and provide empirical evidence to support the development of targeted intervention strategies to improve clinical information security measures.

## METHOD

### Design of Study

A cross-sectional online survey.

### Population and Research Sample

The study population consisted of mainland Chinese nurses and was conducted using convenience sampling. The present study adhered to the Strengthening the Reporting of Observational Studies in Epidemiology (STROBE) statement and checklist. The convenience sampling method was employed to select 756 clinical nurses from 10 public hospitals ranked above the second level in five cities of the Ningxia Hui Autonomous Region (Yinchuan, Shizuishan, Guyuan, Wuzhong and Zhongwei) during November-December 2024. According to the principle of sample size estimation in multifactorial research, with 16 independent variables considered and accounting for a 20% margin for invalid samples^(15)^, a sample size of 200 to 400 was deemed necessary; ultimately, 756 participants were included in the study.

### Inclusion Criteria

Inclusion criteria encompassed individuals aged 18 years and above, possessing a nursing practice certificate, with a minimum of one year of clinical nursing experience, and providing informed consent for voluntary participation.

### Exclusion Criteria

Exclusion criteria excluded nurses from administrative and non-clinical departments, as well as interns and nurses undergoing advanced training.

### Data Collection Tools

Designed by the research team following a review of pertinent literature, the survey comprises demographic variables such as gender, age, marital status, cultural level, hospital grade, title, job level, clinical position, department, employment method, years of work, engagement in clinical teaching, participation in in-house or external information security training programs, involvement in hospital information technology initiatives, and attendance in information security training sessions organized by the hospital or department.

### Information Security Attitude Questionnaire (ISA-Q) for Clinical Nurses

The ISA-Q scale, developed^(16)^ in 2022 and subsequently adapted to Chinese by scholars^(17)^, is primarily utilized to evaluate nurses’ attitudes toward patients’ information security. This scale comprises 6 dimensions with a total of 30 entries: environmental control (3 entries), maintenance of equipment stability (4 entries), information access restriction (4 entries), systematic work (8 entries), enhancement of professional responsibility (6 entries), and continuous education and training (6 entries). Responses to the scale items are rated on a 4-point scale, with 1 representing “completely disagree” and 4 representing “completely agree”. The total score ranges from 30 to 120, with higher scores indicating higher levels of nurses’ attitudes toward information security. The Chinese version of the ISA-Q scale has a Cronbach’s α coefficient of 0.870 and a retest reliability coefficient of 0.884. In the current study, the Cronbach’s α coefficient was determined to be 0.842.

### Nursing Information Competency Rating Scale

The nursing information competence scale, developed^(18)^ through systematic theoretical research, is designed to evaluate the information competence of clinical nursing staff. The scale includes five dimensions: nursing information operation ability, nursing information management ability, nursing information awareness, computer operation ability, and computer software management ability, totaling 32 entries. Responses on the scale are rated on a 5-point Likert scale, ranging from “not at all compliant” to “fully compliant”, with scores of 1 to 5 and a total score of 32 to 160. Higher scores indicate greater nursing information competence among the nurses. The scale has a Cronbach’s α coefficient of 0.719 to 0.947 and a content validity index (CVI) of 0.947. In the present study, the Cronbach’s α coefficient was found to be 0.781.

### Ethical Consideration

This study was approved by the Ethics Committee (approval number: KYLL-2023-0188). Prior to the commencement of the study, all participants received a comprehensive explanation of the research project and were required to provide informed consent. Data collection started only after participants demonstrated a full understanding of the study’s objectives, methodology, and expected collaboration. In addition, the study was conducted in strict accordance with the Declaration of Helsinki.

### Data Collection

An electronic survey using the Star Questionnaire platform was employed for data collection, with researchers liaising with the designated personnel at each participating hospital. Upon obtaining consent from the hospitals, a QR code was disseminated to the nursing departments within the respective institutions. Subsequently, the nursing departments distributed the QR code to individual departments, enabling nurses to complete the electronic questionnaire. The preamble of the questionnaire delineated the study’s content, outlined precautions to be observed by the participants, emphasized the voluntary nature of participation, and required informed consent prior to questionnaire completion. To ensure questionnaire quality, mandatory response options were enforced to facilitate prompt completion and prevent item omission. The survey utilized a non-nominal approach, allowing each participant to submit the questionnaire only once to uphold completeness and prevent duplicate responses. Additionally, measures were implemented to exclude blatantly repetitive responses and questionnaires with response durations of less than 180s.

### Data Analysis

Data were analyzed using IBM SPSS Statistics (Version 27.0). Continuous variables that were not normally distributed are presented as medians with interquartile ranges (P25, P75). The Mann-Whitney U test and the Kruskal-Wallis H test were applied for between-group comparisons. Categorical variables are described as frequencies (percentages), and differences were assessed using rank-sum tests for ordinal data. The relationship between information security attitudes and nursing informatics competence was examined using Spearman’s rank-order correlation.

Multiple linear regression was performed to identify independent predictors of information security attitudes. Variables showing statistical significance (P < 0.05) in univariate analyses were initially entered into the model. To ensure model adequacy, the following steps were undertaken: (1) assumptions of linearity, normality, and homoscedasticity were verified through residual analysis; (2) multicollinearity was assessed using variance inflation factors (VIF), with a VIF < 5 considered acceptable; (3) a stepwise selection procedure was applied to retain only the most relevant predictors, justified by the exploratory nature of this analysis; and (4) regression coefficients are reported with their 95% confidence intervals. All categorical variables were explicitly coded (e.g., training participation: yes = 1, no = 0) and entered as dummy variables where appropriate. Statistical significance was set at P < 0.05 for all tests.

## RESULTS

In this survey, out of the initial 850 questionnaires collected, 94 were excluded due to reasons such as insufficient completion time, irregular responses, or inaccuracies. As a result, 756 valid questionnaires were included, leading to a valid recovery rate of 88.94%. Out of the 850 clinical nurses surveyed, 730 were female (95.6%), 419 participants fell within the 26–35 years category (55.4%), 611 individuals held a bachelor’s degree or higher (80.8%), 308 participants held the designation of nurse practitioner (40.7%), and 270 were at the N2 nursing position level (35.7%). Additionally, 218 respondents reported having 6–10 years of nursing experience (28.8%). Among the participants, 554 individuals were employed by tertiary healthcare institutions (73.3%), 434 had engaged in clinical teaching (57.3%), 681 had participated in information security training courses, either internally or externally (90.1%), 534 were engaged in hospital information technology initiatives (70.6%), and 695 had participated in information security training organized by their hospitals or departments (91.9%). The results are summarized in Table 1.

**Table 1:** General information on clinical nurses; Clinical nurses’ attitudes towards information security univariate analysis results (n = 756, M (P25, P75)) – Yinchuan, Ningxia, China, 2024.

Si. No.	Variables	N	Percentage (%)	ISA-Q	Z/H	P
1	**Gender**					
	Male	26	3.44	89.00 (84.75, 103.25)	1.511	0.131
	Female	730	95.56	96.00 (87.00, 104.00)		
2	**Age Stratification**					
	18~25	43	5.69	99.00 (89.00, 106.00)	22.661	< 0.001
	26~35	419	55.42	99.00 (88.00, 105.00)		
	36-45	217	28.7	90.00 (85.00, 101.00)		
	46~60	77	10.19	89.00 (85.00, 101.00)		
3	**Marriage**					
	Single	96	12.7	101.50 (89.25, 107.00)	2.000	< 0.001
	Married	634	83.86	94.00 (86.75, 104.00)		
	Other	26	3.44	93.00 (86.50, 103.50)		
4	**Educational Background**					
	College	145	19.18	94.00 (87.00, 103.50)	0.981	0.621
	Bachelor’s degree	608	80.42	95.50 (87.00, 105.00)		
	Postgraduate	3	0.4	85.50 (83.00, 88.00)		
5	**Hospital Level**					
	Secondary Hospital	202	26.72	87.00 (83.00, 92.00)	11.116	0.000
	Tertiary Hospital	554	73.28	99.00 (89.00, 105.00)		
6	**Title**					
	Nurse	106	14.02	99.50 (89.00, 106.00)	19.715	< 0.001
	Nurse Practitioner	308	40.74	97.50 (87.00, 105.00)		
	Charge Nurse	288	38.1	91.00 (86.00, 102.00)		
	Associate Chief Nurse or above	56	7.41	91.50 (87.00, 104.00)		
7	**Job level**					
	N0	69	9.13	100.00 (88.00, 108.50)	31.254	< 0.001
	N1	136	17.99	99.00 (89.00, 106.00)		
	N2	270	35.71	97.00 (87.00, 104.00)		
	N3	228	30.16	90.00 (85.25, 101.00)		
	N4	53	7.01	90.00 (85.00, 101.00)		
8	**Clinical Position**					
	Clinical Nurse	583	77.12	95.00 (87.00, 104.00)	0.193	0.908
	Responsibility Team Leader	95	12.57	97.00 (86.00, 104.00)		
	Head Nurse	78	10.32	94.50 (87.75, 104.25)		
9	**Department**					
	Internal Medicine	307	40.61	96.00 (87.00, 105.00)	7.408	0.388
	Surgery	172	22.75	93.00 (85.00, 103.00)		
	Gynecology	51	6.75	98.00 (89.00, 102.00)		
	Pediatrics	32	4.23	97.50 (87.25,105.00)		
	Emergency Department	39	5.16	99.00 (88.00, 105.00)		
	Operating Room	32	4.23	89.00 (86.00, 103.75)		
	Intensive Care Unit	66	8.73	92.00 (85.00, 104.00)		
	Outpatient	57	7.54	96.00 (88.00, 106.50)		
10	**Employment Type**					
	Contract	598	79.1	97.00 (87.00, 105.00)	13.237	0.001
	Permanent staff	140	18.52	90.50 (85.25, 100.00)		
	Other	18	2.38	89.50 (85.50, 106.50)		
11	**Work experience grouping**					
	0–5	148	19.58	100.50 (89.00, 106.00)	18.203	0.001
	6–10	218	28.84	96.00 (86.00, 105.00)		
	11~15	183	24.21	93.00 (87.00, 104.00)		
	16~20	90	11.9	91.00 (84.25, 102.75)		
	21~44	117	15.48	93.00 (87.00, 102.50)		
12	**Whether to participate in clinical teaching**					
	Yes	434	57.41	96.00 (87.00, 105.00)	0.021	0.983
	No	322	42.59	88.00 (83.00, 97.00)		
13	**Have you participated in any information security training courses?**					
	Yes	681	90.08	97.00 (87.00, 105.00)	–4.569	< 0.001
	No	75	9.92	91.00 (86.00, 102.25)		
14	**Have you participated in hospital informatization construction?**					
	Yes	534	70.63	97.00 (88.00, 105.00)	–2.796	0.005
	No	222	29.37	91.00 (86.00, 102.25)		
15	**Does the hospital or department offer training related to information security?**					
	Yes	695	91.93	96.00 (87.00, 105.00)	–5.084	< 0.001
	No	61	8.07	87.00 (82.00, 94.50)		

The aggregate information security attitude score for the 756 clinical nurses was determined to be 95.00 (87.00, 104.00), surpassing the theoretical median of the scale total score (75), indicating a moderate to high level of information security attitude. The overall score rate, derived from the conversion of the total score and individual dimension scores, was calculated at 79.16%. The dimension-specific scores were as follows: environmental control, 66.67%; continual education and training, 66.67%; maintenance of equipment stability, 75%; information access restriction, 75%; work systematization, 81.25%; and promotion of professional responsibility, 83.33%. The results are detailed in Table 2.

**Table 2:** Clinical nurses' information security attitude scores (n = 756, M (P25, P75)) – Yinchuan, Ningxia, China, 2024.

Project	Score range	Scores by dimension	Each entry is equally divided	Scoring rate (%)
Environmental Control	3~12	8.00 (700, 9.00)	2.67 (2.33, 3.00)	66.67
Maintain Equipment Stability	4~16	12.00 (11.00, 13.00)	3.00 (2.75, 3.25)	75.00
Information Access Restriction	4~16	12.00 (11.00, 14.00)	3.00 (2.75, 3.50)	75.00
Work Systematically	8~32	26.00 (23.00, 29.00)	3.25 (2.88, 3.63)	81.25
Enhance Professional Responsibility	6~24	20.00 (18.00, 23.00)	3.33 (3.00, 3.83)	83.33
Continuous Education and Training	6~24	16.00 (15.00, 18.00)	3.20 (3.00, 3.60)	66.67
Total Score of Clinical Nurses' Attitude towards Information	30~120	95.00 (87.00, 104.00)	3.17 (2.90, 3.47)	79.16

The research outcomes revealed that age, marital status, hospital level, title, position level, employment form, years of work experience, participation in in-hospital or out-of-hospital information security training courses, involvement in hospital informationization, and engagement in information security training conducted by the hospital or department significantly influenced clinical nurses’ attitudes toward information security, with all differences being statistically significant (P < 0.05). These findings are presented in Table 1.

The total score of Nursing Information Competence Scale was 128.00 (117.27,145.00), as presented in Table 3. Spearman correlation analysis results indicated a positive correlation between the clinical nurses’ ISA-Q scores and all dimensions of Nursing Information Competence (r = 0.519, all P < 0.05), as displayed in Table 4.

**Table 3:** Nursing information competency scores (n = 756, M (P25, P75)) – Yinchuan, Ningxia, China, 2024.

Project	Score range	Scores for all dimensions	Each entry is equally divided
Nursing Information Awareness	4~20	16.00 (14.00, 18.00)	4.00 (3.50, 4.50)
Computer Operation Ability	8~40	32.00 (30.00, 38.00)	4.00 (3.75, 4.75)
Computer Software Management Ability	5~25	20.00 (15.00, 25.00)	4.00 (4.00, 5.00)
Nursing Information Operation Ability	3~15	14.00 (12.00, 15.00)	4.67 (4.00, 5.00)
Nursing Information Management Ability	12~60	47.00 (40.00, 51.00)	3.92 (3.33, 4.25)
Total Score of Nursing Information Ability	30~160	128.00 (117.00.00, 142.00)	4.00 (3.66, 4.44)

**Table 4:** Correlation between clinical nurses' information security attitude scores and nursing information competency scores (n = 756, r-value) – Yinchuan, Ningxia, China, 2024.

Project	ISA-P	1	2	3	4	5	6
Total Score of Nursing Information Competence	0.519^**^	0.155^**^	0.390^**^	0.397^**^	0.459^**^	0.495^**^	0.431^**^
Nursing Information Awareness	0.433^**^	0.074^*^	0.357^**^	0.342^**^	0.392^**^	0.406^**^	0.350^**^
Computer Operation Ability	0.402^**^	0.138^**^	0.289^**^	0.347^**^	0.357^**^	0.365^**^	0.345^**^
Computer Software Management Ability	0.451^**^	0.104^**^	0.298^**^	0.357^**^	0.421^**^	0.434^**^	0.392^**^
Nursing Information Operation Ability	0.426^**^	0.072^*^	0.309^**^	0.301^**^	0.383^**^	0.409^**^	0.376^**^
Nursing Information Management Ability	0.436^**^	0.174^**^	0.349^**^	0.317^**^	0.361^**^	0.418^**^	0.342^**^

IAS-P: Overall Score of Clinical Nurses' Attitude; 1: Environmental Control; 2: Maintenance Equipment Stability; 3: Information Access Restriction; 4: Work Systematic; 5: Enhance Professional Responsibility; 6: Continuous Education and Training.

*: P < 0.05.

**: P < 0.01.

The total score of clinical nurses’ information security attitude was used as the dependent variable, with statistically significant variables (P < 0.05) identified in the univariate and correlation analyses serving as the independent variables in the multiple stepwise regression analysis. The variable assignment method is as follows: the independent variables were assigned by means of this: age was grouped into 18–25 (=1), 26–35 (=2), 36–45 (=3), and 46–60 (=4); marital status as unmarried (=1), married (=2), and divorced/widowed (=3); hospital level as secondary (=1) and tertiary (=2); professional title as nurse (=1), senior nurse (=2), supervisor nurse (=3), and associate chief nurse (=4); position level from N0 to N4 (1–5); employment type as contract (=1), formal (=2), and other (=3); years of work as 1–5 (=1), 6–10 (=2), 11–15 (=3), 16–20 (=4), and ≥21 (=5). Participation in information security training (inside/outside hospital), involvement in hospital informatization, and participation in hospital/department training were all coded as yes = 1, no = 2. The total nursing informatics competency score was entered as a continuous variable.

The results showed that hospital level, years of work experience, participation in information security-related training organized by the hospital or department, and nursing information competence were determined as the primary influencing factors impacting clinical nurses’ information security attitudes (all P < 0.05), collectively explaining 32.9% of the total variance, as demonstrated in Table 5.

**Table 5:** Multifactorial analysis of factors influencing clinical nurses' attitudes towards information security in the Yinchuan – Ningxia, China, 2024.

Variables	B	SD	β	t	P	VIF	CI	
①	56.482	3.592		15.726	0.000		49.431	63.532
②	–0.923	0.453	–0.063	–2.036	0.042	1.089	–1.814	–0.033
③	4.974	0.830	0.201	5.991	0.000	1.269	3.344	6.603
④	–3.838	1.214	–0.096	–3.162	0.002	1.027	–6.22	–1.455
⑤	0.244	0.020	0.413	12.213	0.000	1.287	0.205	0.283

①constant; ②Age; ③Hospital Level; ④Whether the hospital or department offers information security related training; ⑤Nursing Information Competency.

Notes: R^2^ = 0.332, adjusted R^2^ = 0.329, F = 93.508, P < 0.001.

## DISCUSSION

This study aimed to examine the present state of information security attitudes among clinical nurses and investigate the factors influencing these attitudes. The findings indicated that the overall information security attitude score of 756 clinical nurses was 95.00 (87.00, 104.00), surpassing the theoretical median of 75 and reflecting a moderately high level. However, this score was lower than the findings reported in a survey on the information security attitudes of Korean nurses^(16)^, indicating a need for further improvements in the information security attitudes of clinical nurses. The observed discrepancies may be attributed to variations in geographical and cultural contexts, with the Korean nursing information security education system being relatively advanced, and the societal importance placed on personal privacy fostering a cultural milieu that cultivates positive attitudes towards information security among Korean nurses. This study highlighted the dimensions of professional responsibility and work systematization, which received scores of 83.33% and 81.25%, respectively, suggesting that the clinical nurse population generally exhibits a robust sense of risk prevention and standardized operational practices. This trend could be intricately connected to the enduring emphasis within the medical sector on a culture of accountability and standardized process management.

Nevertheless, the scores for both the environmental control and continuous education and training components were merely 66.67%, indicating the subsequent issues: (1) Inadequate management of the physical environment: observations revealed instances where certain departments lacked medical terminals with automatic locking screens and where mobile devices were stored in an unorganized manner, indicating a lack of diligence in the daily oversight of physical security measures. Safeguarding the physical environment is essential for the protection of hospital computer network systems^(19)^. Studies suggest that deficiencies in physical security measures can heighten the risk of information breaches, potentially compromising the quality of nursing services and jeopardizing patient privacy and safety^(20)^. Hospitals are advised to enhance the management of the physical environment, optimize spatial layout, isolate hardware, and improve measures like data backup to fortify the foundation of information security. (2) Ineffectiveness of training mechanisms: This challenge may stem from the prevalent focus of hospital information security training on theoretical concepts, often overlooking practical implementation and emergency simulations. Consequently, clinical nurses may lack adequate response skills during actual information security incidents. Nursing administrators are advised to enhance the practical aspects and emergency simulation components of the training programs. Furthermore, refining the training strategies and content is essential to ensure quality and effectiveness, ultimately raising the overall level of information security attitudes.

In this study, a positive correlation was identified between nursing information competence and clinical nurses’ information security attitudes (P < 0.05). Similar to the findings^(21)^, it was observed that nursing information competence significantly enhances the information security attitudes of clinical nurses. Nursing information competence encompasses a blend of knowledge, skills, and attitudes that align with the requisite level of nursing practice and are manifest in diverse nursing information-related endeavors^(22)^. Based on the knowledgeattitude-behavior theoretical framework, it can be learned that the improvement of nursing information competence can help to enhance the information security awareness of clinical nurses, enabling them to adeptly recognize information leakage risks and effectively mitigate unsafe practices in nursing, such as unauthorized access, password reuse, and transmission of sensitive information under the public network. In clinical nursing practice, clinical nurses with strong nursing information competence exhibit adeptness in utilizing nursing information systems for the efficient and precise input of patient data, as well as for the effective organization, analysis, and processing of patient information. This proficiency requires not only information management skills to uphold the accuracy, comprehensiveness, and usability of information but also a favorable stance on information security to uphold the security of patient information against subjective vulnerabilities^(23)^. Building upon this foundation, clinical nurses leverage advanced information technology means, such as data encryption and access control measures, to further strengthen the protection of patient privacy, thereby effectively mitigating the risk of information breaches. Moreover, nurses with higher nursing information competence usually receive more systematic education and training, which not only refines their information processing and application proficiencies but also fortifies their commitment to information security. Therefore, nursing administrators should pay attention to the cultivation of clinical nurses’ nursing information competence, aiming at enhancing it through actions such as the strengthening of information security education and training, the provision of information technology support, and the implementation of other strategies, thereby fostering a favorable shift in their information security mindset.

The findings of this study indicated a significant influence of hospital classification on the clinical nurses’ information security attitudes (P < 0.05). Notably, clinical nurses in tertiary hospitals exhibited higher information security attitude scores compared to those in secondary hospitals, aligning with the findings^(24)^. Nurses in tertiary hospitals demonstrated more positive attitudes towards information security, potentially attributed to the concentration of high-quality educational resources, human resources, and medical information technology in these facilities. Tertiary hospitals typically implement more stringent training management systems and standardized frameworks, along with superior resources, providing clinical nurses with enhanced information security training opportunities. Moreover, they typically have more comprehensive information security management systems and regulatory frameworks, fostering a more stringent information security environment for clinical nurses, thus enhancing their comprehension and emphasis on information security, cultivating a more positive stance towards this domain. Secondary hospitals are advised to acknowledge the significance of information technology development, enhance investment in information security, and refine the information security management system. Improvements in information security training for clinical nurses are recommended to boost their competencies and capabilities, empowering them to adeptly address information security challenges.

The results of this study demonstrate that age is a significant factor affecting clinical nurses’ attitudes toward information security (P < 0.05). Nurses aged 25 and below, along with those aged 26 to 35, exhibited the highest scores regarding attitudes toward information security, with the subsequent highest scores observed among nurses aged 36 to 45. This indicates that younger clinical nurses demonstrate more robust attitudes towards information security, aligning with the findings of Liu et al.^(25)^. Research suggests that this is predominantly due to the digital transformation of nursing education systems and the technological advancement within the professional environment^(26)^. Contemporary generations of nurses undergo comprehensive information technology training prior to employment. Their advanced educational credentials and fundamental IT expertise facilitate their quicker comprehension of information security protocols. Simultaneously, the regular application of information systems in frontline environments reinforces security practices within daily routines, creating a normalized pathway for development where ‘usage equates to learning’. Consequently, younger nurses, having been raised in digital environments, demonstrate heightened technological proficiency and learning adaptability, which increases their propensity to cultivate positive attitudes toward information security. However, senior nurses may demonstrate reduced security awareness and behavioral compliance due to factors such as the digital divide, heightened cognitive demands, and incompatible training methods^(27)^. This disparity not only affects individual conduct but may also introduce vulnerabilities into the departmental security culture through behavioral modeling and the transmission of risks within team collaborations. Therefore, age should be regarded not simply as a demographic factor but as a fundamental criterion for designing stratified training approaches. It is advisable for nursing managers to implement tiered training programs tailored to the age categories of clinical nurses, thereby establishing a differentiated training framework. For nurses aged 35 and under, emphasis should be directed towards advanced threat response, data security management, and emergent technology risks. For nurses aged 36 and older, structured step-by-step guidance, scenario-based case analyses, and individualized mentoring should be utilized to strengthen core operational competencies and risk awareness skills. Particularly for senior nurses, it is essential to develop accessible training resources, including visual learning materials and voice-assisted tools, to minimize technical obstacles. Experienced clinical nurses necessitate increased care and attention, in addition to structured support systems such as dedicated technical support centers and clinical information mentors. This mitigates their technological concerns and encourages the enduring adoption of secure practices.

The results of this study demonstrate that engagement in information security training provided by hospitals or departments is a significant factor affecting clinical nurses’ attitudes toward information security (P < 0.05). This indicates that participation in such training constitutes a vital avenue for improving clinical nurses’ awareness of information security^(28)^. The fundamental process of training involves improving nurses’ capacity to recognize and address potential risks through contextualized learning and cognitive restructuring. Purely theoretical instruction produces limited outcomes, whereas practical training that includes real clinical cases, simulation exercises, and mentorship feedback markedly enhances knowledge transfer and behavioral retention. Further investigation indicates that training content should extend beyond fundamental operational topics to include advanced modules such as privacy ethics, legal compliance, social engineering prevention, and security incident reporting procedures, consequently establishing a comprehensive framework for information security literacy. Research conducted by Rose and Kass^(29)^ suggests that clinical nurses are often susceptible to malware infections resulting from improper computer utilization during work, possibly due to insufficient cybersecurity training. Furthermore, research indicates that trained clinical nurses have a certain level of awareness of information security, are proficient in basic information system operation techniques, and possess fundamental skills for managing patient information security^(30)^. Furthermore, the effectiveness of training demands ongoing assessment utilizing multidimensional metrics, including knowledge test scores, detection rates in simulated assaults, rates of clinical security incident reports, and the frequency of operational errors within information systems. This facilitates a closed-loop management approach, shifting the focus from ‘training completion’ to ‘behavioral change’. This emphasizes that nursing administrators must effectively utilize institutional resources to proactively execute information security education and training. Assembling expert teams to design comprehensive, multi-level clinical intervention programs for nursing information security will establish strategic safeguards and lead to the development of comprehensive, competencybased curricula for information security instruction. It is advised that healthcare institutions establish interdisciplinary teams to collaboratively elaborate modular training programs encompassing technical (e.g., identity authentication, mobile device security, malware protection), behavioral (e.g., password management, data sharing protocols, incident reporting procedures) components and legal/ethical considerations (e.g., patient privacy regulations, case studies on ethical decisionmaking) and establish a comprehensive multidimensional evaluation and feedback system to measure training effectiveness. Methods such as behavioral observation, scenario-based assessments, and peer review may be implemented, alongside objective behavioral analysis utilizing information system log data to observe shifts in clinical nurses’ attitudes and to refine training requirements, promoting a supportive organizational environment for information security. Management should explicitly integrate information security into nursing quality assessment frameworks and establish incentive mechanisms to encourage secure behaviors.

## LIMITATIONS OF THE STUDY

This study sought to evaluate the attitudes toward information security and the factors influencing them among clinical nurses in the Ningxia region. Nevertheless, several limitations must be recognized. Initially, reliance on an online survey may have limited participant diversity, potentially affecting the generalizability of the findings to the broader Chinese nursing population. Second, the cross-sectional design limits the scope of variable analysis and the ability to draw causal inferences. Future research should employ longitudinal methodologies to gain more comprehensive insights. Third, dependence on self-reported measures may lead to social desirability bias, potentially resulting in inflated assessments of security attitudes. Fourth, the regional preponderance of the sample and the absence of multi-center data may restrict the generalizability of the findings. Ultimately, organizational-level confounders were not systematically controlled, and multiplecomparison adjustments were not implemented, potentially compromising the robustness of the findings. Future research should aim to broaden sample diversity, employ mixed- methods, integrate objective assessments, and implement more rigorous modeling approaches to further substantiate and extend these findings.

## CONCLUSION

Clinical nurses exhibited a moderate overall attitude toward information security, with notably deficient performance in environmental control and continuous training. Key factors influencing the outcome encompassed age, hospital level, previous training participation, and nursing informatics proficiency.

To enhance attitudes toward information security, the following measures are advised:

- Enhance environmental security measures and associated infrastructure.
- Develop specialized training programs tailored to the age demographics of nursing staff.
- Deliver specialized technological assistance to strengthen nursing informatics proficiency.
- Concentrate training initiatives specifically on senior nurses to mitigate reduced security awareness.

## DATA AVAILABILITY

The entire dataset supporting the results of this study was published in the article itself.
