# Supplementary material for: Current status and factors influencing clinical nurses’ attitudes toward information security: a cross-sectional survey
Source: Rev Esc Enferm USP. 2026 Mar 27;60:e20250391. doi: 10.1590/1980-220X-REEUSP-2025-0391en (PMC13030502; doi:10.1590/1980-220X-REEUSP-2025-0391en)
Supplement: Translation

# Translation: Estado actual y factores que influyen en las actitudes de los enfermeros clínicos hacia la seguridad de la información: una encuesta transversal

**DOI:** 10.1590/1980-220X-REEUSP-2025-0391es

**Authors:** Bai Xue, Xue Yurui, Guo Shuping, Ji Shuangdui, Zheng Donglian, Ma Fuzhen, Yin Changqi

**Affiliations:** 1 Ningxia Medical University, School of Nursing, Ningxia, China.; 2 General Hospital of Ningxia Medical University, Department of Nursing, Ningxia, China.; 3 General Hospital of Ningxia Medical University, Department of Cardiac Macrovascular Surgery, Ningxia, China.; 4 General Hospital of Ningxia Medical University, Department of Orthopaedic Trauma, Ningxia, China.

**Keywords:** Enfermeras Clínicas, Informática Aplicada a la Enfermería, Seguridad Computacional, Actitud

## Abstract

**Objetivo:**

Explorar las actitudes predominantes en materia de seguridad de la información entre el personal de enfermería clínica y examinar los factores que influyen en dichas actitudes.

**Método:**

En noviembre y diciembre de 2024, se empleó un método de muestreo por conveniencia para seleccionar a 756 enfermeros clínicos de diez hospitales públicos clasificados en segundo lugar o superior en cinco ciudades de la Región Autónoma de Ningxia Hui como participantes en el estudio. La encuesta utilizó una escala de evaluación de la competencia en materia de información de enfermería, un cuestionario de información general y un cuestionario para evaluar las actitudes del personal de enfermería hacia la seguridad de la información.

**Resultados:**

La puntuación total de la actitud hacia la seguridad de la información de 756 enfermeras clínicas fue (95,0; 87,0–104,0). El análisis de correlación de Spearman indicó una correlación positiva significativa entre la competencia en información de enfermería y la actitud de las enfermeras clínicas hacia la seguridad de la información (r = 0,519; P < 0,05). Se mostraron los principales resultados de la regresión múltiple, con coeficientes, intervalos de confianza, R^2^ ajustado = 0,329 y la lista de predictores retenidos (edad, nivel del hospital, formación ofrecida, alfabetización informacional)

**Conclusión:**

Este estudio reveló un nivel moderadamente alto de actitudes hacia la seguridad de la información entre las enfermeras clínicas. La edad, la falta de formación previa y una menor competencia informática fueron factores clave asociados con actitudes menos favorables. Por lo tanto, se recomienda implementar intervenciones específicas para reforzar aún más la concienciación sobre la seguridad y promover prácticas seguras en todos los entornos de enfermería.

## INTRODUCCIÓN

Con el avance de la tecnología de la información, los hospitales chinos están desarrollando activamente sistemas de información y aplicaciones móviles de salud. Si bien esto ha mejorado la eficiencia del servicio, también ha puesto de relieve los riesgos de seguridad asociados a los datos de los pacientes. Las investigaciones indican que aproximadamente el 70% de las violaciones de datos sanitarios se deben a una concienciación inadecuada sobre la seguridad entre el personal médico^(1)^. Entre 2009 y 2020, un total de 3705 violaciones de datos sanitarios notificadas en todo el mundo afectaron a casi el 81,72% de la población estadounidense^(2)^, lo que pone de relieve la importancia de esta cuestión. Numerosos estudios sugieren que la principal fuente de los problemas de seguridad de la información sanitaria es la falta de concienciación sobre la seguridad y los fallos de comportamiento del personal interno de las instituciones médicas^(3,4)^. La seguridad de la información de enfermería, como elemento fundamental de los sistemas de información sanitaria, abarca una gran cantidad de datos confidenciales sobre la salud personal e información médica esencial. Los incidentes de seguridad no solo plan-tean el riesgo de intensificar los conflictos entre médicos y pacientes, reducir la calidad de la atención de enfermería y afectar negativamente a la experiencia de los pacientes, sino que también pueden provocar un discurso público negativo y poner en peligro la salud y la seguridad de los pacientes^(5,6)^. Las enfermeras clínicas, como profesionales esenciales directamente responsables del manejo, la recolección y la gestión de la información de los pacientes en la práctica clínica, desempeñan un papel fundamental en los marcos de protección de la seguridad de la información^(7,8)^. Su actitud hacia la seguridad de la información demuestra no solo su dedicación individual a los protocolos de seguridad, sino también un impacto significativo en la gestión y la protección de la información confidencial^(9)^. La investigación internacional se ha centrado en las actitudes, los comportamientos y las competencias de las enfermeras en materia de seguridad de la información, examinando las estrategias de prevención de la filtración, la manipulación y la destrucción de la información de enfermería desde el punto de vista del control tecnológico y administrativo^(7)^. Las actitudes de las enfermeras clínicas hacia la seguridad de la información predicen de manera fiable sus comportamientos en materia de seguridad de la información^(9,10)^. Sin embargo, la investigación nacional se encuentra todavía en una fase inicial, centrándose principalmente en las habilidades de gestión de la información, con una investigación limitada sobre el estado actual de las actitudes de las enfermeras clínicas hacia la seguridad de la información y los factores que las influyen. Hasta la fecha, solo se han realizado investigaciones iniciales sobre la concienciación en materia de seguridad de la información del personal sanitario, como el estudio de Guo y Xie^(11)^. Las pruebas existentes sugieren que las enfermeras clínicas suelen mostrar una conciencia inadecuada del riesgo en las prácticas de seguridad de la información, se muestran complacientes con los protocolos de seguridad y participan en comportamientos como el intercambio indiscriminado de cuentas y el no cerrar sesión en los sistemas con prontitud^(12,13)^. Muestran un conocimiento insuficiente de la protección de la información confidencial, lo que da lugar a prácticas no conformes en el almacenamiento y la transmisión de los datos de los pacientes. Además, no reconocen los riesgos de seguridad que plantean las tecnologías emergentes y confían excesivamente en las configuraciones predeterminadas del sistema. Como parte clave de la región occidental de China, Ningxia muestra ciertas deficiencias en la promoción de la concienciación sobre la seguridad de la información de enfermería y en la implementación de los sistemas pertinentes^(14)^. Por lo tanto, evaluar las actitudes actuales hacia la seguridad de la información entre las enfermeras clínicas de esta región y los factores que las influyen tiene una importancia práctica sustancial. Este estudio tiene por objeto evaluar exhaustivamente el estado actual de las actitudes de las enfermeras clínicas hacia la seguridad de la información, analizar los principales factores que influyen en ellas y proporcionar pruebas empíricas que respalden el desarrollo de estrategias de intervención específicas para mejorar las medidas de seguridad de la información clínica.

## MÉTODO

### Diseño del Estudio

Encuesta transversal en línea.

### Población y Muestra de Investigación

La población del estudio estaba formada por enfermeras de China continental y se llevó a cabo utilizando un muestreo por conveniencia. El presente estudio se ajustó a la declaración y la lista de verificación del Strengthening the Reporting of Observational Studies in Epidemiology (STROBE). Se empleó el método de muestreo por conveniencia para seleccionar a 756 enfermeras clínicas de 10 hospitales públicos clasificados por encima del segundo nivel en cinco ciudades de la Región Autónoma de Ningxia Hui (Yinchuan, Shizuishan, Guyuan, Wuzhong y Zhongwei) durante noviembre y diciembre de 2024. De acuerdo con el principio de estimación del tamaño de la muestra en la investigación multifactorial, con 16 variables independientes consideradas y teniendo en cuenta un margen del 20% para muestras no válidas^(15)^, se consideró necesario un tamaño de muestra de 200 a 400; finalmente, se incluyeron 756 participantes en el estudio.

### Criterios de Inclusión

Los criterios de inclusión abarcaban a personas mayores de 18 años, en posesión de un certificado de práctica de enfermería, con un mínimo de un año de experiencia clínica en enfermería y que dieran su consentimiento informado para participar de forma voluntaria.

### Criterios de Exclusión

Los criterios de exclusión excluían a las enfermeras de los departamentos administrativos y no clínicos, así como a las pasantes y enfermeras en formación avanzada.

### Herramientas de Recolección de Datos

Diseñada por el equipo de investigación tras una revisión de la bibliografía pertinente, la encuesta incluye variables demográficas como el género, la edad, el estado civil, el nivel cultural, el grado hospitalario, el título, el nivel laboral, el puesto clínico, el departamento, el método de contratación, los años de trabajo, la participación en la enseñanza clínica, la participación en programas de formación en seguridad de la información internos o externos, la participación en iniciativas de tecnología de la información hospitalaria y la asistencia a sesiones de formación en seguridad de la información organizadas por el hospital o el departamento.

### Cuestionario de Actitud Hacia la Seguridad de la Información (Information Security Attitude Questionnaire – ISA-Q) Para Enfermeros Clínicos

La escala ISA-Q, desarrollada^(16)^ en 2022 y posteriormente adaptada al chino por académicos^(17)^, se utiliza principalmente para evaluar la actitud de las enfermeras hacia la seguridad de la información de los pacientes. Esta escala comprende seis dimensiones con un total de 30 entradas: control del entorno (3 entradas), mantenimiento de la estabilidad de los equipos (4 entradas), restricción del acceso a la información (4 entradas), trabajo sistemático (8 entradas), mejora de la responsabilidad profesional (6 entradas) y educación y formación continuas (6 entradas).

Las respuestas a los ítems de la escala se califican en una escala de 4 puntos, donde 1 representa “totalmente en desacuerdo” y 4 representa “totalmente de acuerdo”. La puntuación total oscila entre 30 y 120, y las puntuaciones más altas indican un mayor nivel de actitud de las enfermeras hacia la seguridad de la información. La versión china de la escala ISA-Q tiene un coeficiente alfa de Cronbach de 0,870 y un coeficiente de fiabilidad de la repetición de la prueba de 0,884. En el presente estudio, se determinó que el coeficiente alfa de Cronbach era de 0,842.

### Escala de Calificación de la Competencia en Información de Enfermería (Nursing Information Competency Rating Scale)

La escala de competencia en información de enfermería, desarrollada^(18)^ a través de una investigación teórica sistemática, está diseñada para evaluar la competencia en información del personal de enfermería clínica. La escala incluye cinco dimensiones: capacidad de manejo de la información de enfermería, capacidad de gestión de la información de enfermería, concienciación sobre la información de enfermería, capacidad de manejo de computadoras y capacidad de gestión de software informático, con un total de 32 entradas. Las respuestas de la escala se califican en una escala Likert de 5 puntos, que va desde “no cumple en absoluto” hasta “cumple totalmente”, con puntuaciones de 1 a 5 y una puntuación total de 32 a 160. Las puntuaciones más altas indican una mayor competencia en información de enfermería entre las enfermeras. La escala tiene un coeficiente alfa de Cronbach de 0,719 a 0,947 y un índice de validez de contenido (content validity index -CVI) de 0,947. En el presente estudio, se encontró que el coeficiente alfa de Cronbach era de 0,781.

### Consideraciones Éticas

Este estudio fue aprobado por el Comité de Ética (número de aprobación: KYLL-2023-0188). Antes del inicio del estudio, todos los participantes recibieron una explicación exhaustiva del proyecto de investigación y se les pidió que dieran su consentimiento informado. La recolección de datos no comenzó hasta que los participantes demostraron que comprendían plenamente los objetivos del estudio, la metodología y la colaboración esperada. Además, el estudio se llevó a cabo en estricta conformidad con la Declaración de Helsinki.

### Recoleción de Datos

Para la recolección de datos se utilizó una encuesta electrónica mediante la plataforma Star Questionnaire, y los investigadores se pusieron en contacto con el personal designado de cada hospital participante. Una vez obtenido el consentimiento de los hospitales, se distribuyó un código QR a los departamentos de enfermería de las respectivas instituciones. Posteriormente, los departamentos de enfermería distribuyeron el código QR a los departamentos individuales, lo que permitió a las enfermeras completar el cuestionario electrónico. En el preámbulo del cuestionario se describía el contenido del estudio, se esbozaban las precauciones que debían observar los participantes, se hacía hincapié en el carácter voluntario de la participación y se exigía el consentimiento informado antes de completar el cuestionario. Para garantizar la calidad del cuestionario, se impusieron opciones de respuesta obligatorias con el fin de facilitar su rápida cumplimentación y evitar la omisión de preguntas. La encuesta utilizó un enfoque no nominal, lo que permitió a cada participante enviar el cuestionario una sola vez para garantizar su exhaustividad y evitar respuestas duplicadas. Además, se implementaron medidas para excluir las respuestas claramente repetitivas y los cuestionarios con duraciones de respuesta inferiores a 180 segundos.

### Análisis de Datos

Los datos se analizaron utilizando IBM SPSS Statistics (versión 27,0). Las variables continuas que no se distribuían normalmente se presentan como medianas con rangos intercuartílicos (P25, P75). Se aplicaron las pruebas U de Mann-Whitney y H de Kruskal-Wallis para las comparaciones entre grupos. Las variables categóricas se describen como frecuencias (porcentajes) y las diferencias se evaluaron utilizando pruebas de suma de rangos para los datos ordinales. Se examinó la relación entre las actitudes hacia la seguridad de la información y la competencia en informática de enfermería utilizando la correlación de rangos de Spearman.

Se realizó una regresión lineal múltiple para identificar los predictores independientes de las actitudes hacia la seguridad de la información. Las variables que mostraron significación estadística (P < 0,05) en los análisis univariados se introdujeron inicialmente en el modelo. Para garantizar la adecuación del modelo, se llevaron a cabo los siguientes pasos: (1) se verificaron los supuestos de linealidad, normalidad y homocedasticidad mediante el análisis de residuos; (2) se evaluó la multicolinealidad utilizando factores de inflación de la varianza (variance inflation factors – VIF), considerándose aceptable un VIF < 5; (3) se aplicó un procedimiento de selección por pasos para conservar solo los predictores más relevantes, justificado por la naturaleza exploratoria de este análisis; y (4) los coeficientes de regresión se informan con sus intervalos de confianza del 95%.

Todas las variables categóricas se codificaron explícitamente (por ejemplo, participación en la capacitación: sí = 1, no = 0) y se introdujeron como variables ficticias cuando fue apropiado. La significación estadística se fijó en P < 0,05 para todas las pruebas.

## RESULTADOS

En esta encuesta, de los 850 cuestionarios iniciales recolectados, se excluyeron 94 por motivos tales como tiempo de cumplimentación insuficiente, respuestas irregulares o inexactitudes. Como resultado, se incluyeron 756 cuestionarios válidos, lo que supuso una tasa de recuperación válida del 88,94%. De las 850 enfermeras clínicas encuestadas, 730 eran mujeres (95,6%), 419 participantes se encontraban en la categoría de 26 a 35 años (55,4%), 611 personas tenían una licenciatura o un título superior (80,8%) 308 participantes tenían la designación de enfermeras practicantes (40,7%) y 270 ocupaban puestos de enfermería de nivel N2 (35,7%). Además, 218 encuestados declararon tener entre 6 y 10 años de experiencia en enfermería (28,8%). Entre los participantes, 554 personas trabajaban en instituciones de atención médica terciaria (73,3%), 434 se habían dedicado a la enseñanza clínica (57,3%), 681 habían participado en cursos de formación sobre seguridad de la información, ya fuera a nivel interno o externo (90,1%), 534 participaban en iniciativas de tecnología de la información hospitalaria (70,6%) y 695 habían participado en cursos de formación sobre seguridad de la información organizados por sus hospitales o departamentos (91,9%). Los resultados se resumen en la Tabla 1.

**Tabla 1:** Información general sobre las enfermeras clínicas; resultados del análisis univariante de las actitudes de las enfermeras clínicas hacia la seguridad de la información (n = 756, M (P25, P75)) – Yinchuan, Ningxia, China, 2024.

Si. No.	Variables	N	Porcentaje (%)	ISA-Q	Z/H	P
1	**Género**					
	Masculino	26	3,44	89,00 (84,75, 103,25)	1,511	0,131
	Femenino	730	95,56	96,00 (87,00, 104,00)		
2	**Grupos etarios**					
	18~25	43	5,69	99,00 (89,00, 106,00)	22,661	< 0,001
	26~35	419	55,42	99,00 (88,00, 105,00)		
	36-45	217	28,7	90,00 (85,00, 101,00)		
	46~60	77	10,19	89,00 (85,00, 101,00)		
3	**Estado Civil**					
	Soltero	96	12,7	101,50 (89,25, 107,00)	2,000	< 0,001
	casado	634	83,86	94,00 (86,75, 104,00)		
	Otro	26	3,44	93,00 (86,50, 103,50)		
4	**Nivel educativo**					
	Universidad	145	19,18	94,00 (87,00, 103,50)	0,981	0,621
	Bachillerato	608	80,42	95,50 (87,00, 105,00)		
	Post graduación	3	0,4	85,50 (83,00, 88,00)		
5	**Nivel del hospital**					
	Hospital secundario	202	26,72	87,00 (83,00, 92,00)	11,116	0,000
	Hospital terciario	554	73,28	99,00 (89,00, 105,00)		
6	**Titulación**					
	Enfermero	106	14,02	99,50 (89,00, 106,00)	19,715	< 0,001
	Técnico en enfermería	308	40,74	97,50 (87,00, 105,00)		
	Enfermero a cargo	288	38,1	91,00 (86,00, 102,00)		
	Enfermero sub jefe o más	56	7,41	91,50 (87,00, 104,00)		
7	**Nivel del trabajo**					
	N0	69	9,13	100,00 (88,00, 108,50)	31,254	< 0,001
	N1	136	17,99	99,00 (89,00, 106,00)		
	N2	270	35,71	97,00 (87,00, 104,00)		
	N3	228	30,16	90,00 (85,25, 101,00)		
	N4	53	7,01	90,00 (85,00, 101,00)		
8	**Posición clínica**					
	Enfermera clínica	583	77,12	95,00 (87,00, 104,00)	0,193	0,908
	Supervisor responsable	95	12,57	97,00 (86,00, 104,00)		
	Enfermero Jefe	78	10,32	94,50 (87,75, 104,25)		
9	**Departamento**					
	Medicina interna	307	40,61	96,00 (87,00, 105,00)	7,408	0,388
	Cirugía	172	22,75	93,00 (85,00, 103,00)		
	Ginecología	51	6,75	98,00 (89,00, 102,00)		
	Pediatría	32	4,23	97,50 (87,25,105,00)		
	Emergencia	39	5,16	99,00 (88,00, 105,00)		
	Sala de Cirugía	32	4,23	89,00 (86,00, 103,75)		
	Unidad de Cuidados Intensivos	66	8,73	92,00 (85,00, 104,00)		
	Ambulatorios	57	7,54	96,00 (88,00, 106,50)		
10	**Tipo de empleo**					
	Contrato	598	79,1	97,00 (87,00, 105,00)	13,237	0,001
	Staff permanente	140	18,52	90,50 (85,25, 100,00)		
	Otro	18	2,38	89,50 (85,50, 106,50)		
11	**Rangos de experiencia laboral**					
	0–5	148	19,58	100,50 (89,00, 106,00)	18,203	0,001
	6–10	218	28,84	96,00 (86,00, 105,00)		
	11~15	183	24,21	93,00 (87,00, 104,00)		
	16~20	90	11,9	91,00 (84,25, 102,75)		
	21~44	117	15,48	93,00 (87,00, 102,50)		
12	**Participa o no en enseñanza clínica**					
	Si	434	57,41	96,00 (87,00, 105,00)	0,021	0,983
	No	322	42,59	88,00 (83,00, 97,00)		
13	**¿Ha participado en algún curso de capacitación sobre seguridad de la información?**					
	Si	681	90,08	97,00 (87,00, 105,00)	–4,569	< 0,001
	No	75	9,92	91,00 (86,00, 102,25)		
14	**¿Ha participado en la informatización de hospitales?**					
	Si	534	70,63	97,00 (88,00, 105,00)	–2,796	0,005
	No	222	29,37	91,00 (86,00, 102,25)		
15	**¿El hospital o departamento ofrece capacitación relacionada con la seguridad de la información?**					
	Si	695	91,93	96,00 (87,00, 105,00)	–5,084	< 0,001
	No	61	8,07	87,00 (82,00, 94,50)		

La puntuación agregada de la actitud hacia la seguridad de la información de las 756 enfermeras clínicas se determinó en 95,00 (87,00–104,00), superando la mediana teórica de la puntuación total de la escala (75), lo que indica un nivel de actitud hacia la seguridad de la información de moderado a alto. La tasa de puntuación global, derivada de la conversión de la puntuación total y las puntuaciones de las dimensiones individuales, se calculó en un 79,16%. Las puntuaciones específicas de cada dimensión fueron las siguientes: control ambiental, 66,67%; educación y formación continuas, 66,67%; mantenimiento de la estabilidad de los equipos, 75%; restricción del acceso a la información, 75%; sistematización del trabajo, 81,25%; y promoción de la responsabilidad profesional, 83,33%. Los resultados se detallan en la Tabla 2.

**Tabla 2:** Puntuaciones de las enfermeras clínicas en cuanto a su actitud hacia la seguridad de la información (n = 756, M(P25,P75)) – Yinchuan, Ningxia, China, 2024.

Proyecto	Rango de puntuación	Puntos por dimensión	Cada entrada dividida igualmente	Tasa de puntuación (%)
Control ambiental	3~12	8,00 (700, 9,00)	2,67 (2,33, 3,00)	66,67
Mantenimiento de estabilidad del equipamiento	4~16	12,00 (11,00, 13,00)	3,00 (2,75, 3,25)	75,00
restricciones al acceso de información	4~16	12,00 (11,00, 14,00)	3,00 (2,75, 3,50)	75,00
Trabajo sistemático	8~32	26,00 (23,00, 29,00)	3,25 (2,88, 3,63)	81,25
Aumento de responsabilidad profesional	6~24	20,00 (18,00, 23,00)	3,33 (3,00, 3,83)	83,33
Educación continua y entrenamiento	6~24	16,00 (15,00, 18,00)	3,20 (3,00, 3,60)	66,67
Puntuación total de la actitud de las enfermeras clínicas hacia la información	30~120	95,00 (87,00, 104,00)	3,17 (2,90, 3,47)	79,16

Los resultados de la investigación revelaron que la edad, el estado civil, el nivel del hospital, el título, el nivel del puesto, la forma de empleo, los años de experiencia laboral, la participación en cursos de capacitación sobre seguridad de la información dentro y fuera del hospital, la participación en la informatización del hospital y la participación en la capacitación sobre seguridad de la información impartida por el hospital o el departamento influían significativamente en las actitudes de las enfermeras clínicas hacia la seguridad de la información, y todas las diferencias eran estadísticamente significativas (P < 0,05). Estos resultados se presentan en la Tabla 1.

La puntuación total de la Escala de Competencia en Información de Enfermería fue de 128,00 (117,27, 145,00), como se muestra en la Tabla 3. Los resultados del análisis de correlación de Spearman indicaron una correlación positiva entre las puntuaciones ISA-Q de las enfermeras clínicas y todas las dimensiones de la Competencia en Información de Enfermería (r = 0,519, todas P < 0,05), como se muestra en la Tabla 4.

**Tabla 3:** Puntuaciones de competencia en información de enfermería (n = 756, M(P25,P75)) – Yinchuan, Ningxia, China, 2024.

Proyecto	Rango de puntuación	Puntos para todas las dimensiones	Cada entrada dividida igualmente
Conocimiento de información de enfermería	4~20	16,00 (14,00, 18,00)	4,00 (3,50, 4,50)
Habilidad para manejar computadoras	8~40	32,00 (30,00, 38,00)	4,00 (3,75, 4,75)
Habilidad para manejar software	5~25	20,00 (15,00, 25,00)	4,00 (4,00, 5,00)
Habilidad para operar información de enfermería	3~15	14,00 (12,00, 15,00)	4,67 (4,00, 5,00)
Habilidad para gestionar información de enfermería	12~60	47,00 (40,00, 51,00)	3,92 (3,33, 4,25)
Puntuación total de habilidad para manejar información de enfermería	30~160	128,00 (117,00,00, 142,00)	4,00 (3,66, 4,44)

**Tabla 4:** Correlación entre las puntuaciones de actitud hacia la seguridad de la información de las enfermeras clínicas y las puntuaciones de competencia en información de enfermería (n = 756, valor r) – Yinchuan, Ningxia, China, 2024.

Project	ISA-P	1	2	3	4	5	6
Puntuación total de competencia en información de enfermería	0,519^**^	0,155^**^	0,390^**^	0,397^**^	0,459^**^	0,495^**^	0,431^**^
Conocimiento de la información de enfermería	0,433^**^	0,074^*^	0,357^**^	0,342^**^	0,392^**^	0,406^**^	0,350^**^
Habilidad para manejar computadoras	0,402^**^	0,138^**^	0,289^**^	0,347^**^	0,357^**^	0,365^**^	0,345^**^
Habilidad para manejar software	0,451^**^	0,104^**^	0,298^**^	0,357^**^	0,421^**^	0,434^**^	0,392^**^
Habilidad para operar información de enfermería	0,426^**^	0,072^**^	0,309^**^	0,301^**^	0,383^**^	0,409^**^	0,376^**^
Habilidad para gestionar información de enfermería	0,436^**^	0,174^**^	0,349^**^	0,317^**^	0,361^**^	0,418^**^	0,342^**^

IAS-P: Puntuación global de la actitud de las enfermeras clínicas; 1: Control ambiental; 2: Estabilidad del equipo de mantenimiento; 3: Restricción del acceso a la información; 4: Trabajo sistemático; 5: Mejora de la responsabilidad profesional; 6: Educación y formación continuas.

*: P < 0,05.

**: P < 0,01.

La puntuación total de la actitud de las enfermeras clínicas hacia la seguridad de la información se utilizó como variable dependiente, y las variables estadísticamente significativas (P < 0,05) identificadas en los análisis univariantes y de correlación sirvieron como variables independientes en el análisis de regresión múltiple por pasos. El método de asignación de variables es el siguiente: las variables independientes se asignaron de la siguiente manera: la edad se agrupó en 18-25 (= 1), 26-35 (= 2), 36-45 (= 3) y 46-60 (= 4); el estado civil en soltero (=1), casado (= 2) y divorciado/viudo (= 3); el nivel del hospital en secundario (= 1) y terciario (= 2); el título profesional en enfermero (= 1), enfermero superior (= 2), enfermero supervisor (= 3) y enfermero jefe adjunto (= 4); el nivel del puesto de N0 a N4 (1-5); tipo de empleo como por contrato (= 1), formal (= 2) y otro (= 3); años de trabajo como 1–5 (= 1), 6-10 (= 2), 11-15 (= 3), 16-20 (= 4) y ≥21 (= 5). La participación en cursos de formación sobre seguridad de la información (dentro/fuera del hospital), la implicación en la informatización del hospital y la participación en cursos de formación del hospital/departamento se codificaron como sí = 1, no = 2. La puntuación total de competencia en informática de enfermería se introdujo como variable continua.

Los resultados mostraron que el nivel del hospital, los años de experiencia laboral, la participación en cursos de formación relacionados con la seguridad de la información organizados por el hospital o el departamento y la competencia en informática de enfermería se determinaron como los principales factores que influyen en las actitudes de las enfermeras clínicas hacia la seguridad de la información (todos P < 0,05), lo que explica colectivamente el 32,9% de la varianza total, como se muestra en la Tabla 5.

**Tabla 5:** Análisis multifactorial de los factores que influyen en las actitudes de las enfermeras clínicas hacia la seguridad de la información en Yinchuan – Ningxia, China, 2024.

Variables	B	SD	β	t	P	VIF	CI	
①	56,482	3,592		15,726	0,000		49,431	63,532
②	–0,923	0,453	–0,063	–2,036	0,042	1,089	–1,814	–0,033
③	4,974	0,830	0,201	5,991	0,000	1,269	3,344	6,603
④	–3,838	1,214	–0,096	–3,162	0,002	1,027	–6,22	–1,455
⑤	0,244	0,020	0,413	12,213	0,000	1,287	0,205	0,283

①constante; ②Edad; ③Nivel del hospital; ④Si el hospital ofrece capacitacion relacionada a la seguridad de la informacion; ⑤Competencias informacionales de enfermería.

Notas: R^2^ = 0,332, R^2^ ajustado = 0,329, F = 93,508, P < 0,001.

## DISCUSIÓN

El objetivo de este estudio era examinar el estado actual de las actitudes hacia la seguridad de la información entre el personal de enfermería clínica e investigar los factores que influyen en dichas actitudes. Los resultados indicaron que la puntuación global de las actitudes hacia la seguridad de la información de 756 enfermeros clínicos fue de 95,00 (87,00, 104,00), superando la mediana teórica de 75 y reflejando un nivel moderadamente alto. Sin embargo, esta puntuación fue inferior a los resultados obtenidos en una encuesta sobre las actitudes hacia la seguridad de la información de las enfermeras coreanas^(16)^, lo que indica la necesidad de seguir mejorando las actitudes hacia la seguridad de la información de las enfermeras clínicas. Las discrepancias observadas pueden atribuirse a las variaciones en los contextos geográficos y culturales, ya que el sistema educativo coreano en materia de seguridad de la información es relativamente avanzado y la importancia que la sociedad concede a la privacidad personal fomenta un entorno cultural que cultiva actitudes positivas hacia la seguridad de la información entre las enfermeras coreanas. Este estudio destacó las dimensiones de la responsabilidad profesional y la sistematización del trabajo, que recibieron puntuaciones del 83,33% y el 81,25%, respectivamente, lo que sugiere que la población de enfermeras clínicas muestra en general un sólido sentido de la prevención de riesgos y prácticas operativas estandarizadas. Esta tendencia podría estar íntimamente relacionada con el énfasis constante del sector médico en una cultura de responsabilidad y gestión de procesos estandarizados.

No obstante, las puntuaciones de los componentes de control ambiental y educación y formación continuas fueron de solo 66,67%, lo que indica los siguientes problemas: (1) Gestión inadecuada del entorno físico: las observaciones revelaron casos en los que ciertos departamentos carecían de terminales médicas con pantallas de bloqueo automático y en los que los dispositivos móviles se almacenaban de forma desorganizada, lo que indica una falta de diligencia en la supervisión diaria de las medidas de seguridad física. La protección del entorno físico es esencial para la protección de los sistemas de redes informáticas de los hospitales^(19)^. Los estudios sugieren que las deficiencias en las medidas de seguridad física pueden aumentar el riesgo de violaciones de la información, lo que podría comprometer la calidad de los servicios de enfermería y poner en peligro la privacidad y la seguridad de los pacientes^(20)^. Se recomienda a los hospitales que mejoren la gestión del entorno físico, optimicen la distribución del espacio, aíslen el hardware y mejoren medidas como la copia de seguridad de los datos para fortalecer la base de la seguridad de la información. (2) Ineficacia de los mecanismos de formación: este reto puede deberse al enfoque predominante de la formación en seguridad de la información hospitalaria en conceptos teóricos, que a menudo pasa por alto la implementación práctica y las simulaciones de emergencia. En consecuencia, el personal de enfermería clínico puede carecer de las habilidades de respuesta adecuadas durante incidentes reales de seguridad de la información. Se recomienda a los administradores de enfermería que mejoren los aspectos prácticos y los componentes de simulación de emergencias de los programas de formación. Además, es esencial perfeccionar las estrategias y el contenido de la formación para garantizar la calidad y la eficacia, lo que en última instancia elevará el nivel general de las actitudes hacia la seguridad de la información.

En este estudio, se identificó una correlación positiva entre la competencia en información de enfermería y las actitudes de las enfermeras clínicas hacia la seguridad de la información (P < 0,05). De manera similar a los hallazgos^(21)^, se observó que la competencia en información de enfermería mejora significativamente las actitudes de las enfermeras clínicas hacia la seguridad de la información. La competencia en información de enfermería abarca una combinación de conocimientos, habilidades y actitudes que se ajustan al nivel requerido de la práctica de enfermería y se manifiestan en diversas actividades relacionadas con la información de enfermería^(22)^. Basándonos en el marco teórico de conocimiento-actitud-comportamiento, se puede deducir que la mejora de la competencia en información de enfermería puede ayudar a mejorar la concienciación sobre la seguridad de la información de las enfermeras clínicas, permitiéndoles reconocer con destreza los riesgos de fuga de información y mitigar eficazmente las prácticas inseguras en enfermería, como el acceso no autorizado, la reutilización de contraseñas y la transmisión de información confidencial en la red pública. En la práctica de la enfermería clínica, las enfermeras clínicas con una gran competencia en información de enfermería demuestran destreza en la utilización de los sistemas de información de enfermería para la introducción eficiente y precisa de los datos de los pacientes, así como para la organización, el análisis y el procesamiento eficaces de la información de los pacientes. Esta competencia requiere no solo habilidades de gestión de la información para mantener la exactitud, la exhaustividad y la usabilidad de la información, sino también una postura favorable a la seguridad de la información para mantener la seguridad de la información de los pacientes frente a vulnerabilidades subjetivas^(23)^. Sobre esta base, las enfermeras clínicas aprovechan los medios avanzados de tecnología de la información, como el cifrado de datos y las medidas de control de acceso, para reforzar aún más la protección de la privacidad de los pacientes, mitigando así de manera eficaz el riesgo de violaciones de la información. Además, las enfermeras con una mayor competencia en información de enfermería suelen recibir una educación y formación más sistemáticas, lo que no solo perfecciona sus habilidades de procesamiento y aplicación de la información, sino que también fortalece su compromiso con la seguridad de la información. Por lo tanto, los administradores de enfermería deben prestar atención al desarrollo de la competencia en información de enfermería de las enfermeras clínicas, con el objetivo de mejorarla mediante acciones como el fortalecimiento de la educación y la formación en seguridad de la información, la prestación de apoyo en tecnología de la información y la implementación de otras estrategias, fomentando así un cambio favorable en su mentalidad respecto a la seguridad de la información.

Los resultados de este estudio indicaron una influencia significativa de la clasificación de los hospitales en las actitudes de las enfermeras clínicas respecto a la seguridad de la información (P < 0,05). Cabe destacar que las enfermeras clínicas de los hospitales terciarios obtuvieron puntuaciones más altas en cuanto a la actitud hacia la seguridad de la información en comparación con las de los hospitales secundarios, lo que concuerda con los resultados^(24)^. Las enfermeras de los hospitales terciarios mostraron actitudes más positivas hacia la seguridad de la información, lo que podría atribuirse a la concentración de recursos educativos, recursos humanos y tecnología de la información médica de alta calidad en estas instalaciones. Los hospitales terciarios suelen implementar sistemas de gestión de la formación más estrictos y marcos estandarizados, junto con recursos superiores, lo que proporciona a las enfermeras clínicas mejores oportunidades de formación en seguridad de la información. Además, suelen contar con sistemas de gestión de la seguridad de la información y marcos normativos más completos, lo que fomenta un entorno de seguridad de la información más estricto para las enfermeras clínicas, mejorando así su comprensión y énfasis en la seguridad de la información y cultivando una postura más positiva hacia este ámbito. Se recomienda a los hospitales secundarios que reconozcan la importancia del desarrollo de la tecnología de la información, aumenten la inversión en seguridad de la información y perfeccionen el sistema de gestión de la seguridad de la información. Se recomienda mejorar la formación en seguridad de la información de las enfermeras clínicas para potenciar sus competencias y capacidades, capacitándolas para abordar con destreza los retos de la seguridad de la información.

Los resultados de este estudio demuestran que la edad es un factor significativo que influye en la actitud de las enfermeras clínicas hacia la seguridad de la información (P < 0,05). Las enfermeras de 25 años o menos, junto con las de 26 a 35 años, obtuvieron las puntuaciones más altas en cuanto a la actitud hacia la seguridad de la información, seguidas de las enfermeras de 36 a 45 años. Esto indica que las enfermeras clínicas más jóvenes muestran una actitud más sólida hacia la seguridad de la información, lo que coincide con las conclusiones de Liu et al.^(25)^. Las investigaciones sugieren que esto se debe principalmente a la transformación digital de los sistemas de educación en enfermería y al avance tecnológico en el entorno profesional^(26)^. Las generaciones actuales de enfermeras reciben una formación integral en tecnologías de la información antes de incorporarse al mundo laboral. Sus credenciales educativas avanzadas y sus conocimientos básicos de TI les permiten comprender más rápidamente los protocolos de seguridad de la información. Al mismo tiempo, la aplicación regular de los sistemas de información en entornos de primera línea refuerza las prácticas de seguridad en las rutinas diarias, creando una vía normalizada para el desarrollo en la que “el uso equivale al aprendizaje”. En consecuencia, las enfermeras más jóvenes, que se han criado en entornos digitales, demuestran una mayor competencia tecnológica y capacidad de adaptación al aprendizaje, lo que aumenta su propensión a cultivar actitudes positivas hacia la seguridad de la información. Sin embargo, las enfermeras más experientes pueden mostrar una menor conciencia de la seguridad y un menor cumplimiento de las normas de comportamiento debido a factores como la brecha digital, las mayores exigencias cognitivas y los métodos de formación incompatibles^(27)^. Esta disparidad no solo afecta a la conducta individual, sino que también puede introducir vulnerabilidades en la cultura de seguridad del departamento a través del modelado del comportamiento y la transmisión de riesgos dentro de las colaboraciones en equipo. Por lo tanto, la edad debe considerarse no solo como un factor demográfico, sino como un criterio fundamental para diseñar enfoques de formación estratificados. Es aconsejable que los directores de enfermería implementen programas de formación por niveles adaptados a las categorías de edad de las enfermeras clínicas, estableciendo así un marco de formación diferenciado. En el caso de las enfermeras de 35 años o menos, se debe hacer hincapié en la respuesta avanzada a las amenazas, la gestión de la seguridad de los datos y los riesgos tecnológicos emergentes. En el caso de las enfermeras de 36 años o más, se debe utilizar una orientación estructurada paso a paso, análisis de casos basados en escenarios y tutorías individualizadas para reforzar las competencias operativas básicas y las habilidades de concienciación sobre los riesgos. En particular, en el caso de las enfermeras de más edad, es esencial desarrollar recursos de formación accesibles, incluidos materiales de aprendizaje visuales y herramientas asistidas por voz, para minimizar los obstáculos técnicos. Las enfermeras clínicas con experiencia necesitan más cuidados y atención, además de sistemas de apoyo estructurados, como centros de asistencia técnica especializados y mentores de información clínica. Esto mitiga sus preocupaciones tecnológicas y fomenta la adopción duradera de prácticas seguras.

Los resultados de este estudio demuestran que la participación en la formación en seguridad de la información impartida por los hospitales o departamentos es un factor significativo que influye en la actitud de las enfermeras clínicas hacia la seguridad de la información (P < 0,05). Esto indica que la participación en dicha formación constituye una vía fundamental para mejorar la concienciación de las enfermeras clínicas sobre la seguridad de la información^(28)^. El proceso fundamental de la formación consiste en mejorar la capacidad de las enfermeras para reconocer y abordar los riesgos potenciales mediante el aprendizaje contextualizado y la reestructuración cognitiva. La enseñanza puramente teórica produce resultados limitados, mientras que la formación práctica que incluye casos clínicos reales, ejercicios de simulación y comentarios de los mentores mejora notablemente la transferencia de conocimientos y la retención de comportamientos. Investigaciones adicionales indican que el contenido de la formación debe ir más allá de los temas operativos fundamentales e incluir módulos avanzados como la ética de la privacidad, el cumplimiento legal, la prevención de la ingeniería social y los procedimientos de notificación de incidentes de seguridad, estableciendo así un marco integral para la alfabetización en materia de seguridad de la información. Las investigaciones realizadas por Rose y Kass^(29)^ sugieren que las enfermeras clínicas suelen ser susceptibles a las infecciones de malware como resultado de un uso inadecuado de las computadoras durante el trabajo, posiblemente debido a una formación insuficiente en materia de ciberseguridad. Además, la investigación indica que las enfermeras clínicas capacitadas tienen un cierto nivel de conciencia sobre la seguridad de la información, dominan las técnicas básicas de operación de sistemas de información y poseen habilidades fundamentales para gestionar la seguridad de la información de los pacientes^(30)^. Además, la eficacia de la capacitación exige una evaluación continua que utilice métricas multidimensionales, incluyendo las calificaciones de las pruebas de conocimientos, las tasas de detección en ataques simulados, las tasas de informes de incidentes de seguridad clínica y la frecuencia de errores operativos dentro de los sistemas de información. Esto facilita un enfoque de gestión de ciclo cerrado, desplazando el enfoque de la “finalización de la capacitación” al “cambio de comportamiento”. Esto pone de relieve que los administradores de enfermería deben utilizar eficazmente los recursos institucionales para ejecutar de forma proactiva la educación y la capacitación en materia de seguridad de la información. La creación de equipos de expertos para diseñar programas de intervención clínica integrales y multinivel para la seguridad de la información en enfermería establecerá salvaguardias estratégicas y conducirá al desarrollo de planes de estudio integrales y basados en competencias para la instrucción en materia de seguridad de la información. Se recomienda que las instituciones sanitarias establezcan equipos interdisciplinarios para elaborar de forma colaborativa programas de capacitación modulares que abarquen componentes técnicos (por ejemplo, autenticación de identidad, seguridad de dispositivos móviles, protección contra malware), conductuales (por ejemplo, gestión de contraseñas, protocolos de intercambio de datos, procedimientos de notificación de incidentes) y consideraciones legales/éticas (por ejemplo, normativas de privacidad de los pacientes, estudios de casos sobre la toma de decisiones éticas), y que establezcan un sistema integral de evaluación y retroalimentación multidimensional para medir la eficacia de la capacitación. Se pueden implementar métodos como la observación del comportamiento, las evaluaciones basadas en escenarios y la revisión por pares, junto con el análisis objetivo del comportamiento utilizando los datos de registro del sistema de información para observar los cambios en las actitudes de las enfermeras clínicas y perfeccionar los requisitos de formación, promoviendo un entorno organizativo que favorezca la seguridad de la información. La dirección debe integrar explícitamente la seguridad de la información en los marcos de evaluación de la calidad de la enfermería y establecer mecanismos de incentivo para fomentar comportamientos seguros.

## LIMITACIONES DEL STUDIO

Este estudio trató de evaluar las actitudes hacia la seguridad de la información y los factores que las influyen entre las enfermeras clínicas de la región de Ningxia. No obstante, hay que reconocer varias limitaciones. En primer lugar, el hecho de basarse en una encuesta en línea puede haber limitado la diversidad de los participantes, lo que podría afectar a la generalización de los resultados a la población de enfermeras china en general. En segundo lugar, el diseño transversal limita el alcance del análisis de variables y la capacidad de extraer inferencias causales. Las investigaciones futuras deberían emplear metodologías longitudinales para obtener una visión más completa. En tercer lugar, la dependencia de medidas autoinformadas puede dar lugar a un sesgo de deseabilidad social, lo que podría dar lugar a evaluaciones exageradas de las actitudes hacia la seguridad. En cuarto lugar, el predominio regional de la muestra y la ausencia de datos multicéntricos pueden limitar la generalización de los resultados. Por último, no se controlaron sistemáticamente los factores de confusión a nivel organizativo y no se aplicaron ajustes de comparación múltiple, lo que podría comprometer la solidez de los resultados. Las investigaciones futuras deberían tener como objetivo ampliar la diversidad de la muestra, emplear métodos mixtos, integrar evaluaciones objetivas e implementar enfoques de modelización más rigurosos para corroborar y ampliar estos hallazgos.

## CONCLUSIÓN

Las enfermeras clínicas mostraron una actitud general moderada hacia la seguridad de la información, con un rendimiento notablemente deficiente en el control del entorno y la formación continua. Los factores clave que influyeron en el resultado fueron la edad, el nivel del hospital, la participación previa en cursos de formación y el dominio de la informática aplicada a la enfermería.

Para mejorar las actitudes hacia la seguridad de la información, se recomiendan las siguientes medidas:

- Mejorar las medidas de seguridad ambiental y la infraestructura asociada.
- Desarrollar programas de formación especializados adaptados a la demografía por edades del personal de enfermería.
- Proporcionar asistencia tecnológica especializada para reforzar la competencia en informática de enfermería.
- Concentrar las iniciativas de formación específicamente en las enfermeras de mayor edad para mitigar la reducción de la concienciación sobre la seguridad.

## DISPONIBILIDAD DE DATOS

Todo el conjunto de datos que apoya los resultados de este estudio fue publicado en el propio artículo.
